# Vortex-Mixing Microfluidic Fabrication of Micafungin-Loaded Magnetite–Salicylic Acid–Silica Nanocomposite with Sustained-Release Capacity

**DOI:** 10.3390/ma17235816

**Published:** 2024-11-27

**Authors:** Doina-Antonia Mercan, Adelina-Gabriela Niculescu, Alexandra Cătălina Bîrcă, Diana-Elena Cristea, Alina Moroșan, Dana-Ionela Tudorache, Bogdan Purcăreanu, Bogdan Ștefan Vasile, Dana Radu, Mihai Alexandru Grigoroscuta, Tony Hadibarata, Dan Eduard Mihaiescu, Alexandru Mihai Grumezescu

**Affiliations:** 1 Department of Science and Engineering of Oxide Materials and Nanomaterials, National University of Science and Technology Politehnica Bucharest, 011061 Bucharest, Romania; antonia.mercan@gmail.com (D.-A.M.); alexandra.birca@upb.ro (A.C.B.); dana.tudorache@upb.ro (D.-I.T.); bogdanpb89@gmail.com (B.P.); hadibarata@curtin.edu.my (T.H.); grumezescu@yahoo.com (A.M.G.); 2 Research Institute of the University of Bucharest—ICUB, University of Bucharest, 050657 Bucharest, Romania; 3 Department of Organic Chemistry, National University of Science and Technology POLITEHNICA Bucharest, 011061 Bucharest, Romania; dianacristea98@yahoo.com (D.-E.C.); alina.morosan@upb.ro (A.M.); danedmih@gmail.com (D.E.M.); 4 BIOTEHNOS SA, Gorunului Rue, No. 3-5, 075100 Otopeni, Romania; 5 Research Center for Advanced Materials, Products and Processes, National University of Science and Technology POLITEHNICA Bucharest, 060042 Bucharest, Romania; bogdan.vasile@upb.ro; 6 National Research Center for Micro and Nanomaterials, National University of Science and Technology POLITEHNICA Bucharest, 060042 Bucharest, Romania; 7 National Institute of Materials Physics, Street Atomistilor 405 A, 077125 Magurele, Romania; dana.radu@infim.ro (D.R.); alex_bebe07@yahoo.com (M.A.G.); 8 Environmental Engineering Program, Faculty of Engineering and Science, Curtin University Malaysia, CDT 250, Miri 98009, Malaysia

**Keywords:** iron oxide, silica, core shell, vortex microfluidic platform, micafungin, antifungal delivery

## Abstract

Iron oxide nanoparticles were synthesized using a vortex microfluidic system and subsequently functionalized with a primary shell of salicylic acid, recognized for its ability to increase the stability and biocompatibility of coated materials. In the second stage, the vortex platform was placed in a magnetic field to facilitate the growth and development of a porous silica shell. The selected drug for this study was micafungin, an antifungal agent well regarded for its effectiveness in combating fungal infections and identified as a priority compound by the World Health Organization (WHO). The resulting nanocomposite system was characterized using various techniques, including Fourier transform infrared spectroscopy (FT-IR), X-ray diffraction (XRD), transmission electron microscopy (TEM), dynamic light scattering (DLS), Brunauer–Emmett–Teller (BET) analysis, UV-Vis spectroscopy, and Fourier transform ion cyclotron resonance mass spectrometry (FT-ICR MS). The synthesis method produced nanoparticles with dimensions of 5–7 nm, highlighting the advantages of the chosen approach. A desorption profile was established using a continuous-flow, UV-Vis analysis system, indicating that the bioactive compound was released slowly; after two hours, approximately 50% of the loaded micafungin was detected in the release medium. Furthermore, the results obtained from the FT-ICR MS analysis provided molecular-level confirmation, thereby supporting the release mechanism of micafungin from the nanosystem.

## 1. Introduction

Knowledge of drug delivery systems is predominant in the medical field, and nanocomposites play a crucial aspect in medication development. Nanocomposites, formed of nanoscale materials, exhibit significance in drug delivery due to particular characteristics attributed to their surface and rheological properties, including the controlled release of therapeutic agents, improving efficacy, and minimizing side effects [1,2,3]. The accurate delivery of medication to the expected site, an essential advantage of nanocomposites, is performed through targeted drug delivery, enhancing efficiency. This precision decreases systemic exposure and allows for lower drug dosages, mitigating potential negative effects [4]. The prominence of accurate medication delivery lies in its promise to transform medical treatment, contributing to a more effective and patient-friendly alternative [5]. Nanocomposites have also been demonstrated to encapsulate, secure, and deliver drugs in particular areas, which allows customized medicine and more successful therapeutic interventions [4,6].

Micafungin is a promising antifungal agent broadly employed for its therapeutic capability in curing fungal infections [7]. The antifungal agent is involved in preventing the synthesis of beta-glucan in the fungal cell wall, destroying its structure and leading to fungal cell death [8]. The stability of micafungin relies on temperature and alkaline conditions, causing the appearance of the degradation product. Another challenge comes from current delivery methods, as its administration generally involves intravenous infusion, limiting the availability and approachability. Furthermore, challenges related to this delivery method, including the potential for infusion-related reactions and the need for medical guidance, indicate the importance of innovative delivery approaches to improve the adoption of micafungin [9]. To address these challenges, some methods could be conducted to give medicine in improved forms for safer and easier delivery. Incorporating three distinct surfaces and creating organic–inorganic hybrid systems are critical for enabling agent release through interactions with biological structures. This design is responsive to various stimuli, such as pH changes, which are particularly relevant in the context of fungal infections [10,11]. The goals were to help patients pursue their medication better and secure the best results from micafungin in treating fungal infections [12].

The magnetite–salicylic acid–silica nanocomposite is an innovative and promising drug delivery approach composed of three elements: a magnetic core, a functional component, and a protective shell [13,14]. The magnetic core allows suitable handling and pointing by external magnetic fields, developing the precision of drug delivery [15,16,17]. Salicylic acid, which has healing properties, assists the nanocomposites to work well [18,19,20]. To utilize the material effectively as a controlled release system, it is essential for iron oxide nanoparticles to be encapsulated in an organic layer. This approach offers two main advantages: First, applying salicylic acid as the initial coating on the magnetic nanoparticles eliminates concerns about oxidation. More importantly, it serves as a stabilizing agent, significantly reducing the tendency for agglomeration [21,22]. Simultaneously, salicylic acid serves a dual role in the design of the developed material, offering anti-inflammatory, antimicrobial, and antifungal properties that enhance its overall effectiveness [23]. The magnetite–salicylic acid–silica nanocomposite exhibits some advantages in controlling drug release, improving drug stability, and assembling therapeutic and diagnostic capabilities [24,25]. This nanocomposite also establishes its potential as an impressive and functional platform for a leading drug delivery application [13].

Vortex-mixing microfluidic technology is an advanced method of creating nanocomposites, using apparent principles to revolutionize the procedure [26,27]. This innovative process provides many benefits to produce nanocomposites. First, the controlled vortex mixing ensures that the nanoparticles of other parts are expanded constantly, resulting in an excellent and more consistent nanocomposite [27]. Third, the microfluidic system has a high surface area compared to its volume, allowing for faster reactions and the improvement of nanocomposites’ efficiency [26].

A sustained-release drug delivery system is essential in medicine because it handles things that conventional drug delivery methods are inefficient at [28,29,30]. Unlike conventional methods that rapidly release medicine, sustained-release systems moderately release medicine over time, resulting in a lower possibility of side effects as a result of modifying the medicine dosage. Additionally, the sustained-release system improves the bioavailability of drugs and optimizes the drug level in the body for a longer period. This system is important in medical situations like chronic disease or conditions requiring long-term medication [4,31].

The study aimed to develop a micafungin-loaded nanocomposite and assess its sustained-release capacity. Focusing on nanotechnology, it seeks to contribute insights into enhanced drug delivery systems for improved therapeutic outcomes.

## 2. Materials and Methods

### 2.1. Materials

The complete synthesis procedure entails the sequential use of various substances, depending on the specific stages of the synthesis method. Consequently, the following materials are essential for the development of the materials relevant to this study: ferric chloride (FeCl_3_) and iron sulfate heptahydrate (FeSO_4_·7H_2_O), purchased from Sigma Aldrich Merck (Darmstadt, Germany); salicylic acid (HOC_6_H_4_COOH), supplied by Atochim Prod (Bucharest, Romania); and sodium hydroxide (NaOH) from Lach-Ner (Tovarni, Czech Republic). Cetyltrimethylammonium bromide (C_19_H_42_BrN) and sodium trisilicate (Na_2_O_7_Si_3_) were also acquired from Sigma Aldrich Merck (Darmstadt, Germany). Acetic acid (CH_3_COOH) and ethanol (C_2_H_6_O), purchased from Emsure Merk Millipore (Darmstadt, Germany), as well as micafungin (C_56_H_71_N_9_O_23_S), from Medichem (Birżebbuġa, Malta), were used in the synthesis process. All of the chemicals used were of analytical grade and did not require any further purification. Ultrapure water was used consistently throughout the synthesis process.

### 2.2. Microfluidic Platform Fabrication

**Figure 1:**
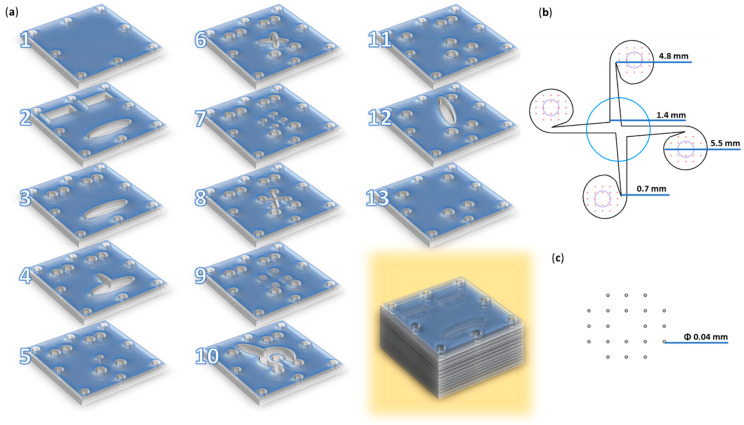
Microfluidic platform configuration. (**a**) Schematic 3D representation of the microfluidic platform: individual layers and overlapped view. (**b**) Overlayed reaction area: blue—channel for iron precursors solution (layer 9); red—channels for salicylic–silica solutions (layer 9); black—vortex mixing chamber and its dimensions (layer 8); purple—collecting channels (layer 7). (**c**) Reactant inlet dimensions (layer 9). Adapted from our previous study [32].

The magnetic nanoparticles, intended to serve as a core that was functionalized with salicylic acid followed by a salicylic acid–silica shell, were specifically designed for synthesis through a microfluidic method utilizing a vortex-type chip. The micromixer was created by overlapping 13 square polymethylmethacrylate pieces (i.e., side = 70 mm) with the laser-cut patterns indicated in Figure 1. A more detailed description of the utilized microreactor is available in our previous study [32].

### 2.3. Nanocomposite Preparation

#### 2.3.1. Fe_3_O_4_-SA NPs—Microfluidic Synthesis Method

The first step in the synthesis procedure for the composite nanometric and complex material focuses on obtaining magnetite nanoparticles functionalized with salicylic acid on their surface, which occurs directly within the vortex microfluidic platform. The required stock solutions were prepared as follows:

The iron precursor solution assumes a 0.01 M solution of FeCl_3_ and a 0.03 M solution of FeSO_4_·7H_2_O, totaling 900 mL of ultrapure water. The second solution involves preparing a 0.5 M NaOH solution in 900 mL of ultrapure water, along with the addition of 2 g of salicylic acid for the direct functionalization of the magnetite nanoparticles. The prepared solutions were concurrently introduced into the vortex-based mixing microfluidic chip using a pump, enabling the co-precipitation of the iron ions at the intersection with the alkaline solution and resulting in the formation of salicylic acid-functionalized magnetite nanoparticles (Fe_3_O_4_-SA NPs). The resulting precipitate was allowed to settle on a strongly magnetic surface, after which, the supernatant was removed. Several washes with ultrapure water were then carried out, and ultimately, the Fe_3_O_4_-SA NPs were dispersed in ultrapure water for further use.

#### 2.3.2. Fe_3_O_4_-SA-SiO_2_ NPs—Microfluidic Synthesis Method

The subsequent step, following the production of core-functionalized nanoparticles composed of magnetite and salicylic acid, entails the formation of an additional silica shell layer. This process also employs a vortex microfluidic approach, utilizing the same chip design and parameters. To further enhance the Fe_3_O_4_-SA NPs’ core-functionalized system with a silica shell, it was necessary to carry out two initial synthesis procedures to prepare the stock solutions for the reaction within the vortex microfluidic platform, as outlined below.

**Figure 2:**
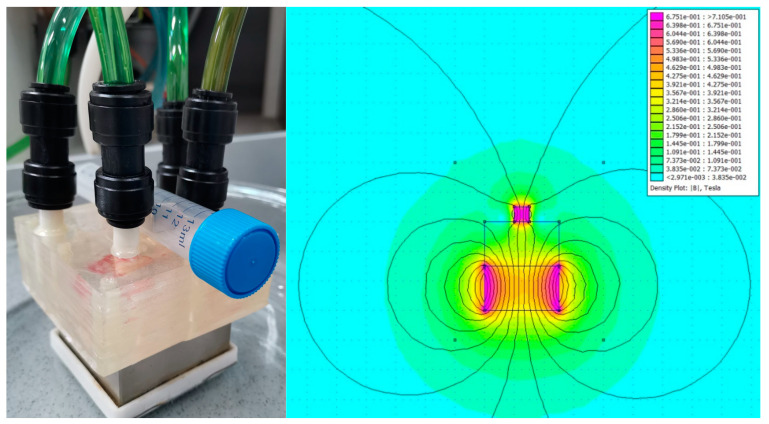
(**left**) The experimental set-up and (**right**) the FEMM model of the magnetic field configuration.

The solution designated as the main core, which has an initial layer of salicylic acid, is prepared by mixing 76.8 mL of Fe_3_O_4_-SA NP dispersion with 90 mL of acetic acid and then adding this mixture to a solution of 5 µM CTAB in a total volume of 3000 mL of ultrapure water. To form the silica shell on the Fe_3_O_4_-SA NPs, a 0.01 M sodium trisilicate solution is used, having a total volume equal to the previously prepared solution, along with the incorporation of 42 g of NaOH during the synthesis process. The stock solutions obtained are used as inputs for the vortex-type microfluidic platform, where they are introduced simultaneously to facilitate the reaction upon contact. Additionally, the microfluidic system is arranged between two neodymium magnets, providing a magnetic induction of 0.3 Tesla in the area where the reactants mix, helping to align the magnetic field for achieving the desired morphology (Figure 2).

#### 2.3.3. Fe_3_O_4_-SA-SiO_2_ NPs—Micafungin Drug Loading

To enhance the targeted use of the Fe_3_O_4_-SA-SiO_2_ nanoparticles produced via a vortex-type microfluidic system, we employed a gradual solvent evaporation technique for the loading process with an antifungal agent. Micafungin was chosen to develop a drug delivery system due to its remarkable efficacy against fungal pathogens. The process entails the gradual evaporation of the solvent from a solution, which enables the solute to precipitate or crystallize. This method of controlled evaporation typically leads to the proper dosage and bioavailability of micafungin throughout the silica shell.

**Figure 3:**
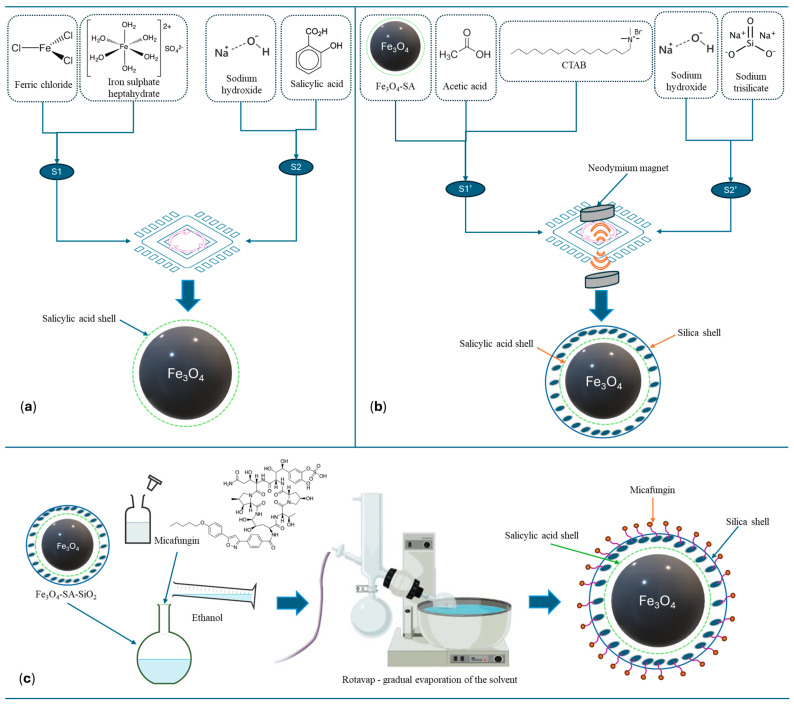
Schematic representation of nanocomposite preparation. (**a**) Microfluidic synthesis method for the production of Fe_3_O_4_-SA NPs. (**b**) Microfluidic synthesis method for the production of Fe_3_O_4_-SA-SiO_2_ NPs. (**c**) Micafungin drug loading onto Fe_3_O_4_-SA-SiO_2_ NPs via gradual solvent evaporation.

A visual overview of the synthesis methods described in Section 2.3.1, Section 2.3.2 and Section 2.3.3 is represented in Figure 3.

### 2.4. Characterization Methods

#### 2.4.1. X-Ray Diffraction—XRD

The crystallinity of the samples was assessed using a PANalytical Empyrean diffractometer (PANalytical, Almelo, The Netherlands), equipped with a hybrid monochromator (2xGe 220) on the incident side and a parallel plate collimator coupled with a PIXcel 3D detector on the diffracted side. The measurements were conducted at room temperature via grazing incidence X-ray diffraction (GIXRD), with an incidence angle of ω = 0.5° over Bragg angles (2θ) ranging from 10° to 80°. The diffractometer utilized Cu Kα radiation with a wavelength of λ = 1.5406 Å, operating at 40 mA and 45 kV.

#### 2.4.2. Fourier Transform Infrared Spectroscopy—FTIR

The FTIR spectroscopy analysis technique was used to obtain relevant information based on the infrared spectra of absorption containing specific functional groups. A Thermo Nicolet 6700 (Thermo Fisher Scientific, Waltham, MA, USA) spectrometer model was used in the H-ATR module with a ZnSe crystal. The data were obtained through the measurements performed in the range of 4000–400 cm^−1^, setting a resolution of 8 cm^−1^ and performing 64 scans per spectrum.

#### 2.4.3. Dynamic Light Scattering—DLS

The dynamic light scattering (DLS) technique was used for particle size distribution using a Nano ZS Zetasizer (Malvern Instruments, Malvern, UK)-type device. The measurements were performed at a spreading angle of 90° and at a temperature of 25 °C. Three data acquisitions were made per sample, resulting in the relevant average diameter and polydispersity index values.

#### 2.4.4. Transmission Electron Microscopy—TEM

The structure and phase composition of the nanomaterials were analyzed using transmission electron microscopy (TEM). The TEM micrographs were acquired on a Tecnai™ G2 F30 S-TWIN electron microscope, equipped with selected area electron diffraction (SAED) from FEI (Hillsboro, OR, USA). The microscope was operated in transmission mode at an accelerating voltage of 300 kV, achieving point and line resolutions of 2 Å and 1.02 Å, respectively. The TEM specimens were prepared by dispersing the nanopowders in ethanol, followed by 15 min of ultrasonic treatment. The sample was then deposited onto a carbon-coated copper grid and allowed to dry at room temperature.

#### 2.4.5. Brunauer–Emmett–Teller—BET

The Brunauer–Emmett–Teller (BET) analysis involved obtaining nitrogen adsorption/desorption isotherms at 77 K over a relative pressure range of p/po = 0.005–1.0, using a NOVA 800 Gas Sorption Analyzer (Anton Paar QuantaTec, Inc., Boyton Beach, FL, USA). Kaomi-type software (Anton Paar Kaomi for Nova version 1.0) was used for data processing. A degassing process was carried out under vacuum at 180 °C for 4 h before actually performing the adsorption measurements. The standard BET equation was applied to calculate the specific surface area. The gas volume, absorbed at a relative pressure of approximately p/po~1, was used to estimate the total pore volume. Additionally, the Barrett–Joyner–Halenda (BJH) model was employed to determine the pore size distribution and mesopore volume from the desorption branch of the isotherm.

#### 2.4.6. Ultraviolet-Visible Spectroscopy

The ultraviolet-visible (UV–Vis) spectroscopy analysis was used to evaluate the release of micafungin from the core-shell, magnetite-based delivery systems. An Evolution 300 double-beam type spectrophotometer (Waltham, MA, USA) was operated to obtain the relevant results. Absorbance values were recorded between 220 and 380 nm, and further data processing was performed using the tools offered by the 4.5.0 VisionPro software.

#### 2.4.7. Fourier Transform Ion Cyclotron Resonance Mass Spectrometry—FT-ICR

The XR FTMS Hybrid System QqFTMS utilizes mass spectrometry with a superconducting magnet, specifically the SolariX XR 15T. The high-resolution mass spectrometry analysis was conducted using a Fourier transform ion cyclotron resonance (FT-ICR) spectrometer, the SolariX XR 15T (Bruker Daltonics, Bremen, Germany). The sample was introduced through direct infusion using negative ESI ionization, with a sample flow rate of 200 µL/h, and a nebulization gas pressure (N_2_) set at 1.2 bar at 190 °C, coupled with a flow rate of 4 L/min. The spectra were acquired over a mass range of 92 to 1500 amu, at a source voltage of 4300 V.

## 3. Results

The nanomaterials obtained were analyzed to determine their physicochemical properties, beginning with the assessment of the functional groups present in the structures of the magnetite-based systems.

**Figure 4:**
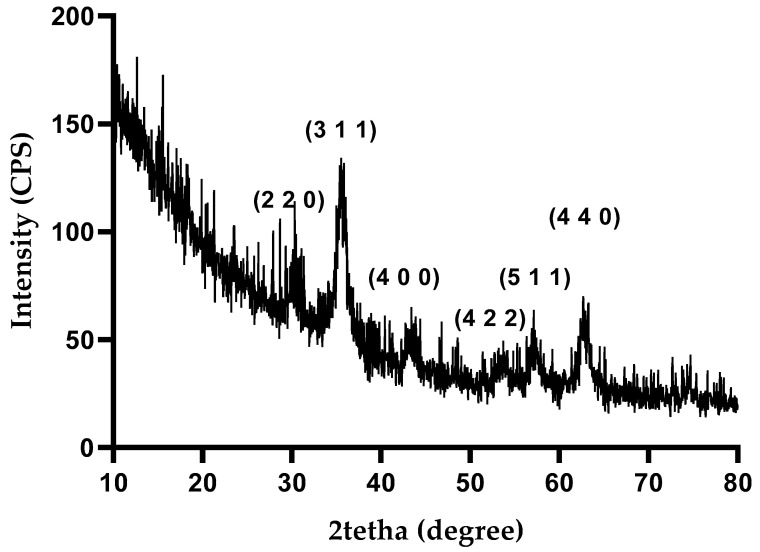
XRD pattern of Fe_3_O_4_-SA-SiO_2_.

The X-ray diffraction (XRD) analysis was conducted to identify the crystalline phases present in the synthesized nanoparticles. Figure 4 shows the diffractogram of Fe_3_O_4_-SA-SiO_2_, where sharp diffraction peaks at 2θ values of 30.31, 35.71, 43.31, 53.90, 57.61, and 62.81 are visible. These peaks, corresponding to scattering from specific planes, denoted by the Miller indices (220), (311), (400), (422), (511), and (440), align well with the characteristic diffraction pattern of magnetite, as referenced by the American Society for Testing Materials (ASTM), sheet code 01-075-160 [33].

**Figure 5:**
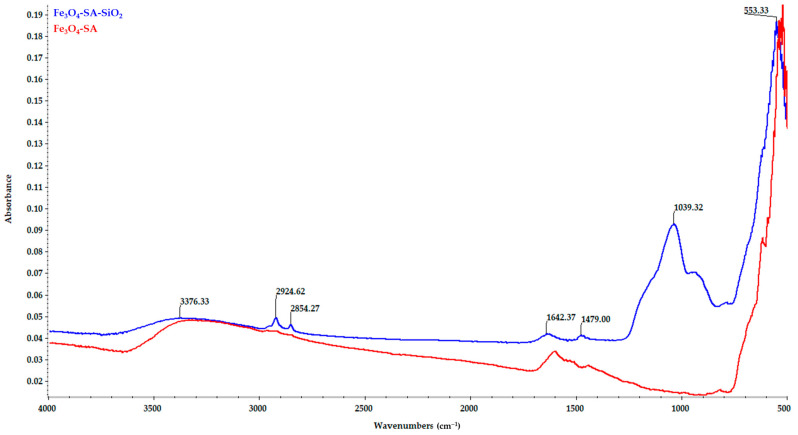
FT-IR spectra—comparisons between Fe_3_O_4_-SA and Fe_3_O_4_-SA-SiO_2_.

The comparative FT-IR spectra of the magnetic nanoparticles Fe_3_O_4_-SA and Fe_3_O_4_-SA-SiO_2_ are presented in Figure 5. The presence of the organic layer coating the magnetic nanoparticles Fe_3_O_4_-SA is proven by the characteristic band for the -OH stretching vibrations at 3322 cm^−1^. The C=O stretching band of the carboxyl group can be observed at 1601 cm^−1^, and the characteristic absorption band for asymmetric stretching vibrations of carboxylate groups can be observed at 1534 cm^−1^. The characteristic band at 821 cm^−1^ evidences the aromatic structure of SA. The O-H bending band from the carboxylic group can be observed at 1442 cm^−1^, and the characteristic band assigned to the Fe-O bond vibration can be observed at 523 cm^−1^. The presence of a silica shell for the Fe_3_O_4_-SA-SiO_2_ nanoparticles is proven by the characteristic absorption for asymmetric Si-O-Si stretching vibrations at 1039 cm^−1^ and the Si-OH bond band at 934 cm^−1^. The characteristic band of magnetite can be observed at 553 cm^−1^, corresponding to the Fe-O bond vibration.

**Figure 6:**
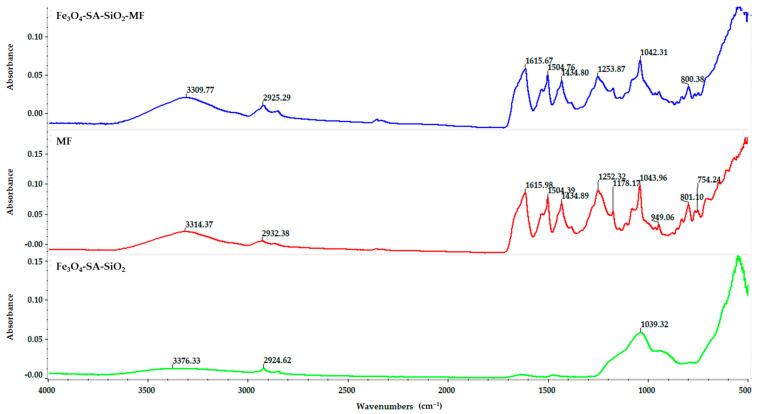
FT-IR spectra—comparisons between Fe_3_O_4_-SA-SiO_2_-micafungin, micafungin, and Fe_3_O_4_-SA-SiO_2_.

The effective loading of the nanostructured material with the bioactive compound micafungin was evidenced in the FT-IR spectrum. The FT-IR spectrum of pure micafungin compared to the FT-IR spectra of Fe_3_O_4_-SA-SiO_2_-micafungin and Fe_3_O_4_-SA-SiO_2_ are shown in Figure 6. The broad band at 3314 cm^−1^ is attributed to the following: the -OH stretching vibration and the stretching vibration of CH at 2932 cm^−1^ and 2866 cm^−1^, respectively. The signals detected at 1615, 1504, and 1434 cm^−1^ belong to the stretching of C=C aromatic bonds. The FT-IR spectrum of the Fe_3_O_4_-SA-SiO_2_-micafungin nanostructured material does not show significant changes, and the characteristic band of magnetite corresponding to the vibration of the Fe-O bond can be observed at 547 cm^−1^.

**Figure 7:**
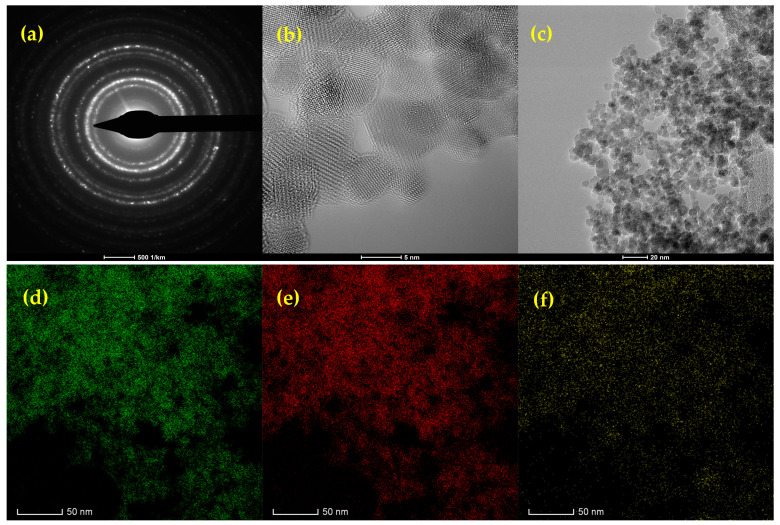
(**a**) SAED pattern, (**b**) high-resolution TEM image, (**c**) bright-field TEM image, and (**d**–**f**) HRSTEM-EDS elemental maps of Fe_3_O_4_-SA-SiO_2_ nanoparticles. Elemental mapping displays the distribution of (**d**) Fe, (**e**) O, and (**f**) Si.

Figure 7 presents a TEM micrograph of Fe_3_O_4_-SA-SiO_2_, illustrating regions with dispersed nanoparticles, as well as areas of homo-aggregation, where identical Fe_3_O_4_ particles (5–7 nm) cluster together. This homo-aggregation likely results from intermolecular forces, such as Van der Waals interactions, causing the nanoparticles to attract each other and form larger clusters, rather than remaining uniformly dispersed. The selected area electron diffraction (SAED) ring pattern, shown in Figure 7a, corresponds to the (220), (311), (400), (422), (511), and (440) lattice planes of magnetite. This pattern confirms the presence of a single crystalline magnetite phase, which aligns well with the findings from the XRD analysis [34].

The HRSTEM-EDS elemental mapping of the Fe_3_O_4_-SA-SiO_2_ sample provides a detailed view of the spatial distribution of iron (Fe), oxygen (O), and silicon (Si) within the nanocomposite. The green map shows Fe uniformly dispersed, which originates from the Fe_3_O_4_ core, suggesting a well-dispersed core structure without significant aggregation. The red map displays the distribution of oxygen, present both in the Fe_3_O_4_ core and in the SiO_2_ shell. This widespread oxygen signal indicates the successful integration of the oxide materials in both the core and shell.

The yellow map, which represents Si, confirms the presence of a silica layer around the Fe_3_O_4_-SA nanoparticles. The Si distribution around the Fe signal indicates a well-formed and continuous silica coating, which likely enhances the stability and dispersibility of the nanoparticles.

**Figure 8:**
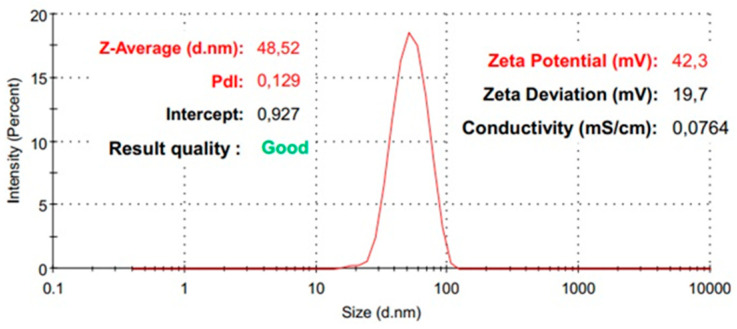
The distribution of the average hydrodynamic diameter of Fe_3_O_4_-SA.

**Figure 9:**
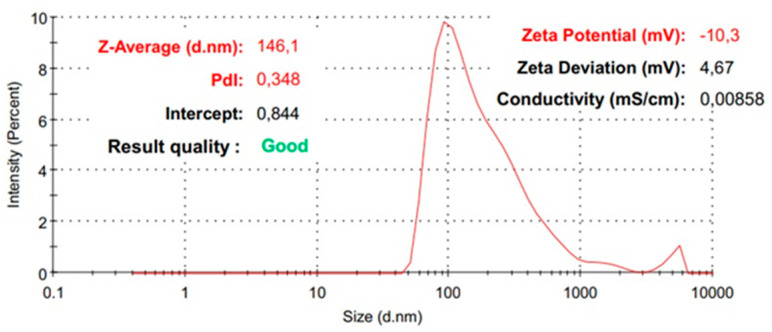
The distribution of the average hydrodynamic diameter of Fe_3_O_4_-SA-SiO_2_.

Further, the samples were analyzed using the DLS technique in order to obtain information related to the hydrodynamic diameter, the polydispersity index, and the zeta potential. Considering the goal of obtaining a silica shell on a magnetite core, the hydrodynamic diameter is expected to change progressively with the improvement of the nanocomposite system. The DLS analysis proves that the obtained Fe_3_O_4_-SA nanoparticles show an increase in the hydrodynamic diameter by shell formation with silica from 48 nm to 146 nm (Figure 8 and Figure 9). The shape of the average distribution profile of the hydrodynamic diameter of the nanostructured system with a silica shell shows a secondary distribution. The nanostructured systems show excellent dimensional homogeneity, recorded for Fe_3_O_4_-SA, with a very low polydispersity index of 0.129. The addition of a silica shell on the magnetite core indicates a modest modification of the polydispersity index, with a value of 0.348, which still indicates good dimensional homogeneity with only slight variations. Remarkable results were obtained in terms of colloidal stability, correlating the zeta potential value of 42 mV for Fe_3_O_4_-SA with excellent dispersion and non-sedimentation properties. On the other hand, the silica shell’s presence is observed through the modification of the zeta potential of Fe_3_O_4_-SA-SiO_2_ to −10 mV. This modification is normal, since the particles’ surface is no longer correlated with the characteristics of magnetite, which generally have a positive zeta potential. The results clearly indicate that the presence of a silica shell leads to a negative surface charge. However, the zeta potential measurement results indicate the modest, but adequate, colloidal stability of Fe_3_O_4_-SA-SiO_2_.

**Figure 10:**
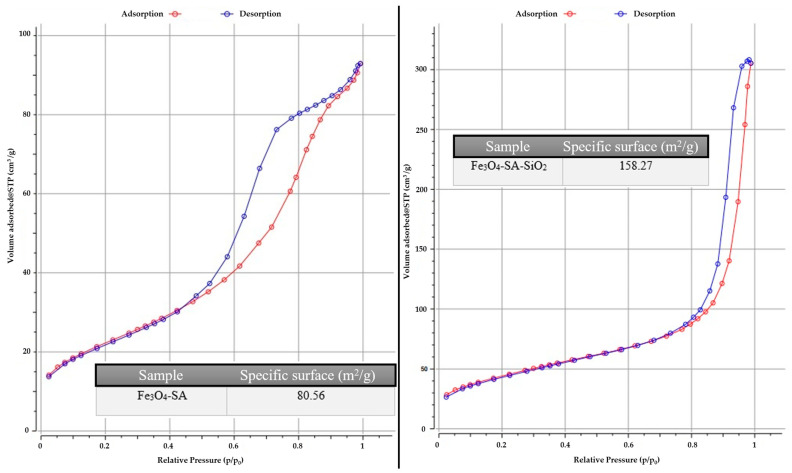
The N_2_ adsorption/desorption isotherm for the Fe_3_O_4_-SA and Fe_3_O_4_-SA-SiO_2_ samples.

Considering the complexity of generating a silica shell on a magnetic core functionalized with salicylic acid under microfluidic vortex-type synthesis conditions, it is necessary to evaluate properties such as the specific surface area, size, and volume of the pores. These characteristics were determined by measuring the gas adsorption on the material’s surface and subsequently applying the BET model for the analysis (Figure 10). Therefore, the results obtained for the Fe_3_O_4_-SA sample show the values of the specific surface of 80.56 m^2^/g, with a large specific surface that is correlated with the fine powder and the presence of a small nanoparticle agglomeration. On the other hand, the result obtained for the sample Fe_3_O_4_-SA-SiO_2_ indicates a value of 158.27 m^2^/g, correlated with a very high specific surface. Supplementary information regarding pore size and pore volume was collected for each sample. The Fe_3_O_4_-SA sample has values for pore size, dBJH, of 7.0320 nm and pore volume, Vp, of 0.1355 cm^3^/g, and for the Fe_3_O_4_-SA-SiO_2_ sample, both values increase, reaching values for pore size, dBJH, of 22.127 nm and pore volume, Vp, of 0.4674 cm^3^/g. This is due to the creation of the silica shell on the magnetic core, which substantially improves the intended application in terms of the adsorption process of the antifungal drug. The isotherm shapes indicate a mesoporous-associated type of isotherm, recorded for Fe_3_O_4_-SA-SiO_2_.

Given the application discussed in this study, the use of micafungin involves an initial phase, in which it is released from the core-shell nanostructured complex, followed by its biological activity in eliminating harmful fungal species. The UV-Vis analysis technique was employed to assess the quantitative release capacity of micafungin from the nanostructured support, beginning with the evaluation of various concentrations, 3.33, 17.33, 33.60, 67.60, and 100.66 mg L^−1^, and the plotting of the calibration curve (Figure S1). The maximum absorbance value of micafungin at 270 nm was used for calibration curve plotting and the subsequent quantification. A correlation coefficient value of 0.9998 was obtained, demonstrating the linearity of the quantification range used.

The sustained-release profile of micafungin was obtained by recording the minute-by-minute absorbance readings in kinetic mode for a monitoring period of about 2 h (138 min). This analysis method allowed the observation of the release profile, which was progressive and dependent on time (Figure S2).

**Figure 11:**
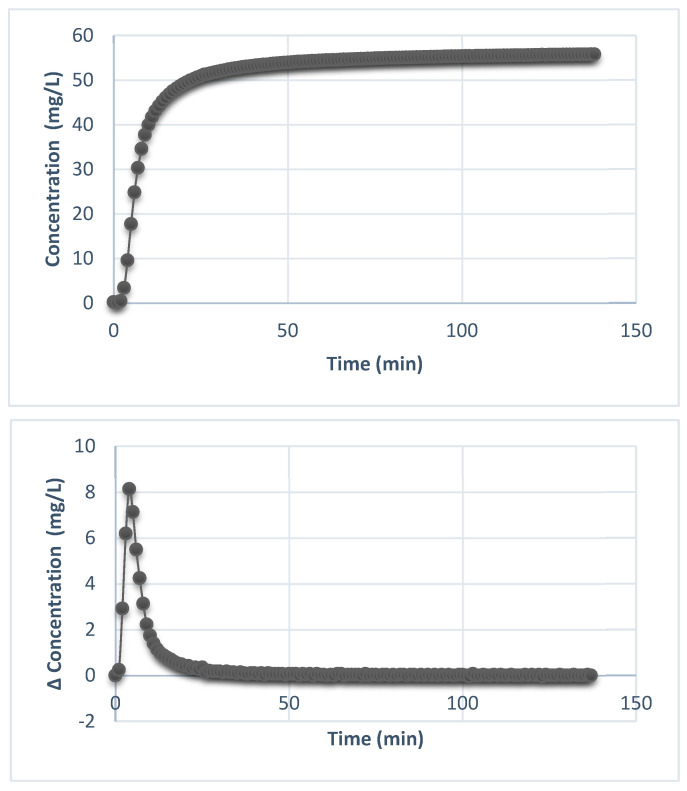
The variation of the micafungin concentration over time and the variation in the incremental release of micafungin (the difference in concentration vs. time).

As can be seen in Figure 11, approximately 40% of the adsorbed material was rapidly released in the first 40 min. When micafungin is incorporated into silica-shell pores, a small part of the drug molecules can be released more rapidly because the permeable structure enables water or biological fluids to enter and dissolve the drug at an increased rate. This initial fast release is identified as a burst that can provide immediate therapeutic benefits, particularly in situations where rapid antifungal effects are required. However, after the rapid-burst release, a constant concentration was recorded until the end of the experiment. This is associated with the time-controlled release of micafungin, which corresponds to the purpose of the intended application and, at the same time, demonstrates the ability of the core-shell nanostructured system to release the drug with an antifungal effect under a certain profile.

It is observed that after 2 h, only 50% of the loaded material had been desorbed, recommending this material for sustained-release applications as it was capable of maintaining an adequate concentration of the active substance at the site of infection for an extended period and allowing the sustained action of micafungin in treating the area of interest.

**Figure 12:**
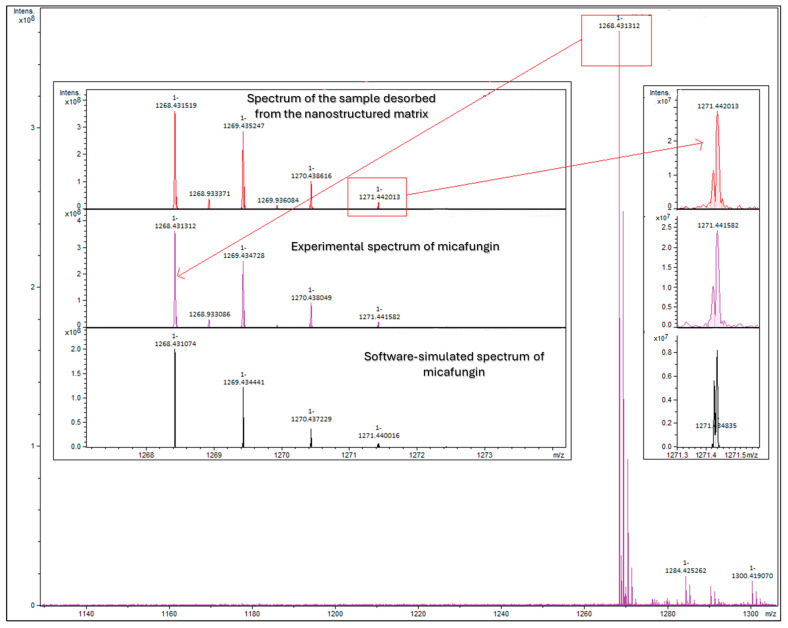
The FT-ICR MS confirmation of the sustained desorption of micafungin from the nanostructured matrix.

Additional confirmation of the sustained desorption of micafungin from the nanostructured core-shell design was performed through the FT-ICR MS analysis of the sample containing the micafungin that was released after sustained desorption for 2 h or, more exactly, 138 min (Figure 12). The obtained mass spectrum highlights the agreement between the isotopic profile of the prediction that was based on the molecular formula and the micafungin standard of the resulting solution after desorption. An additional check for the ([M + H]^+^ + 3) peak highlights the similarity of the isotopomer peaks in the isotopic fine structure.

## 4. Discussion

The purpose of this study involved developing and testing a core-shell-type system with applications in releasing an antifungal agent and targeting harmful strains, all of which were synthesized through an innovative microfluidic platform method with vortex-type geometry. There are advantages to microfluidic synthesis, especially with the utilized vortex-type geometry. For example, the contact between the reactant solutions is high, the mixing efficiency is significantly improved, and also a continuous circulation of the fluids is supported. The reaction time is faster due to the homogeneity of the fluids circulating through the chip. A uniform, consistent distribution of the reaction solutions is encountered, which reduces the formation of concentration gradients. There is also adaptability and control regarding the synthesis parameters correlated with the final properties of the material of interest [27,35,36,37]. Moreover, the formation of a core-shell, nanostructured system directly on the microfluidic platform involves the shell encapsulation of a core that is continuously generated in a controlled manner, which leads to morphological and dimensional uniformity that is also based on the ratio of the core to the shell fluids, which is, in turn, related to the thickness of the shell layer [38,39,40].

Besides the advantages of the resulting nanomaterials, the utilization of the microfluidic platform also provides benefits in terms of costs and the ease of synthesis. The employed microfluidic vortex system provides a streamlined, efficient process that minimizes reaction times and simplifies production operations, reducing the multi-step synthesis to a single, continuous-flow process. This synthesis innovation reduces nanoparticle production’s technical complexity, potentially facilitating scalability and reproducibility. Moreover, utilizing the vortex microfluidic system could reduce the overall resource requirements due to its efficient mixing and reaction capabilities, minimizing waste and optimizing material use. As it involves reduced reagent mounts, minimal chemical waste, and low energy demands, the proposed synthesis route is eco-friendly, adhering to sustainable practices.

The specific advanced analysis techniques revealed the features of the synthesized core-shell nanocomplex. In this sense, the Fe–O fingerprint region was distinct in all of the FTIR spectra patterns in the 523–553 cm^−1^ range, confirming the formation of the magnetic nano-core. The formation of the SiO_2_ shell was evidenced by the vibrational bands at 934 cm^−1^ and 1093 cm^−1^ and the asymmetric vibration of the Si-O-Si and Fe-O-Si stretching vibrations [41,42,43,44]. Xia Chen et al. [45] developed a core-shell system based on silica-coated iron oxide nanoparticles under the reverse microemulsion method, which also included the silica surface’s modifying process. Rui-Feng Guo et al. [44] prepared and tested a core-double-shell, iron oxide-based system that included a silica first layer and a magnesium borate second layer with applications in the removal of methylene blue and copper from contaminated water. Hossein Hosseinzadeh et al. [46] published an article regarding the amino-functionalized, β-cyclodextrin silica iron oxide core-shell nanocarrier for doxorubicin. The characteristic functional groups that clearly indicate the silica shell’s formation on the magnetic core that were observed in their FTIR results were similar to our results. Due to the vortex design of the microfluidic platform, the well-known long duration of the reaction and the multi-step executions were eliminated by using this novel, adapted method accompanied by the main goal of obtaining small-size nanoparticles with a modest, but enclosed, shell. Moreover, surface modifications are no longer required to have an improved feature when it comes to including the drug of interest in the silica pores for developing a drug delivery system. Generally, the carboxyl group C=O is found near the wavenumber of 1690 cm^−1^, but the Fe_3_O_4_-SA-SiO_2_ spectrum presented a shift of C=O to 1634 cm^−1^. This shift may be associated with the interaction of the silica shell with the core nanoparticles’ surface [41,47]. Micafungin was easily identified in the FTIR spectra obtained from the functionalized samples due to the aromatic chemical structures identified by the absorption bands at 1615, 1504, and 1434 cm^−1^ [48,49].

The silica shell prevents the iron oxide core from oxidating and offers chemical stability, which also decreases the particle agglomeration tendency. The DLS measurement results provided information that strongly suggests the successful shelling of the magnetite core. The increased value of the hydrodynamic diameter is directly correlated with the formation of a silica-covering system. The mean value registered for the Fe_3_O_4_-SA nanoparticles was 48 nm, and it was 146 nm for the Fe_3_O_4_-SA-SiO_2_ nanoparticles. The zeta potential behavior of the measured samples exhibited a significant modification, associated with the silica shell’s formation. The change from the positive zeta potential to the negative zeta potential appeared because of the silanol groups found on the silica surface [50,51]. The benefits of using a microfluidic synthesis approach are demonstrated again. Stijn Smulders et al. [52] synthesized SiO_2_-Fe_3_O_4_ core-shell nanoparticles in two steps, involving the co-precipitation and the Stöber method, revealing a value of 1150.6 nm for hydrodynamic diameter and −4.6 mV for the zeta potential. Marcela Slováková et al. [53] published a research article that involved the utilization of a Fe_3_O_4_@SiO_2_ core-shell trypsin immobilization in protein digestion. They prepared three types of Fe_3_O_4_@SiO_2_ with an NH_2_ group surface modification by varying the (3-aminopropyl) triethoxysilane amount in the synthesis method. Moreover, the hydrodynamic diameter was measured in distilled water, Tween 20, and phosphate buffer, with values in the range of 100–200 nm.

It is well known that the microfluidic synthesis of nanoparticles has, at first thought, the goal of very small nanoparticle sizes. SEM micrographs of the magnetic core-shell system can reveal the core particle formation with small dimensional characteristics that present a disparity shell of silica. Considering the small size of the particles, their individual morphology is quite complicated to identify, but certain areas are identified at high magnifications, in which spherical structures with a high predisposition to agglomeration can be distinguished. Baoliang Zhang et al. [54] synthesized Fe_3_O_4_@SiO_2_ hollow nanoparticles by the hydrothermal method, at first, for the core formation, then tetraethyl orthosilicate was added and left to react for the silica shell formation. They obtained a particularly interesting morphology of the particles with sizes between 150–200 nm. Seham S. Alterary et al. [55] also approached a classic method of synthesizing magnetite nanoparticles with silica shells, obtaining morphologies of the core-shell system suitable for the purpose and with dimensions of approximately 53 nm. Moreover, the silica shell presented some surface roughness, and the differences between the contrast of the darker areas associated with the core and the whiter areas associated with silica indicated that there were particles with either a thicker or a thinner formed shell. This may also be a consequence of particle agglomeration due to their small size. The EDS spectra supported the formation of silica shells by identifying Si and O as constituent elements in the analyzed sample.

Regarding the BET result, the core-shell system indicated the inclusion of silica in the structure on the surface of the iron oxide nanoparticles, especially through the different values of the specific surface area obtained for the Fe_3_O_4_-SA sample and for the Fe_3_O_4_-SA-SiO_2_ sample. Moreover, the pore size values for both of the samples were different due to the presence of the silica shell, which also indicated the size types of the pores; in all cases, the mesoporous characteristic was evidenced. The isotherm type is associated with the type IV-IUPAC Classification of Adsorption isotherms, which is also characteristic of the mesopores’ nature [53,56,57]. The two samples’ specific surface characteristics and porous properties revealed a distinct correlation between the Fe_3_O_4_-SA-SiO_2_ sample and its favorable properties for the adsorption and desorption of certain substances, such as micafungin. Additionally, the small pore size restricts the movement of adsorbed molecules, prolonging their contact time with the mesoporous material surface and, ultimately, enhancing the antifungal feature [58].

The final goal of the study consisted of the impregnation of micafungin into the surface and the pore network of the silica shell and achieving its controlled release over time. This approach aimed to facilitate a single treatment process that provides long-term efficacy. It is essential for micafungin to act over an extended duration to ensure consistency and effective fungal combat. However, a controlled release is needed, which supports the long action period and minimizes any adverse conditions that may impact its use. The UV-Vis result indicated the kinetic behavior of the micafungin in contact with a liquid medium for a period of time. The slow profile over time after an initial release due to the first impact with a liquid environment was confirmed; subsequently, a constant on the concentration expressed in mg/L was maintained throughout the experiment. The efficacy of micafungin was demonstrated in a study made by Javad Naderi et al. [59], who adopted an antifungal surface-coating concept using micafungin on a silicon substrate. The study results indicated that micafungin has an impressive antifungal activity against *Candida albicans* and could be reutilized at least five times, even considering the exhaustive washing process, lengthy soaking periods, and elevated temperatures, which normally highly reduce the concentration of micafungin correlated with the antifungal activity.

The FT ICR analysis result demonstrated that the molecular confirmation of micafungin correlated with its release. The precise detection of the equipment is associated with the interaction of micafungin with the silica matrix without any structural changes. The FT-ICR MS results indicated a stable release of micafungin, as evidenced by the consistent peak observed at 1268.431312 *m*/*z*, which precisely corresponds to its intact molecular structure.

Thus, the micafungin-loaded magnetite–salicylic acid–silica nanocomposite demonstrated a sustained-release profile, with an initial rapid release followed by a gradual, controlled release. This behavior is desirable, being beneficial for prolonged therapeutic action, reduced dosing frequency, and potentially improved patient compliance. Moreover, in comparison to conventional drug delivery systems, the developed nanocomposite offers targeted release capabilities while maintaining effective drug concentrations over time at the infection site.

## 5. Conclusions

In order to manage the new age of resistant microorganisms, it is essential to create innovative therapies that effectively combat these infections. In this context, traditional methods that incorporate antifungal agents into specific materials and conventional synthesis techniques for these materials have been bypassed. This study utilized a vortex-type microfluidic approach to create a complex core-shell system with various properties, the primary one being antifungal activity, which positively influenced the physicochemical properties of the resulting material. The analysis results demonstrated an increase in the silica shell surrounding the magnetic core, complemented by an additional organic layer of salicylic acid, providing a more comprehensive understanding of the assembly. Additionally, the incorporation of micafungin into the silica pore network and its subsequent release behavior was examined and validated, confirming the achievement of the intended objectives. Thus, the microfluidically produced nanosystem is poised to fulfill its primary function while offering multiple advantages related to antifungal activity, along with a verified, comprehensive plan from application to treatment completion.

## Data Availability

The original contributions presented in the study are included in the article/Supplementary Material, further inquiries can be directed to the corresponding author.

## Supplementary Materials

The following supporting information can be downloaded at https://www.mdpi.com/article/10.3390/ma17235816/s1. Figure S1: Micafungin standards with concentrations between 3.33 and 100.66 mg L^−1^ and the corresponding plotted calibration curve; Figure S2: The kinetic time-dependent sustained desorption profile of micafungin recorded in the equipment software.
