# Supplementary material for: Vortex-Mixing Microfluidic Fabrication of Micafungin-Loaded Magnetite–Salicylic Acid–Silica Nanocomposite with Sustained-Release Capacity
Source: Materials (Basel). 2024 Nov 27;17(23):5816. doi: 10.3390/ma17235816 (PMC11642680; doi:10.3390/ma17235816)
Supplement: Supplementary file 1 [file materials-17-05816-s001.zip › materials-3280902-supplementary.pdf]

Supplementary Materials

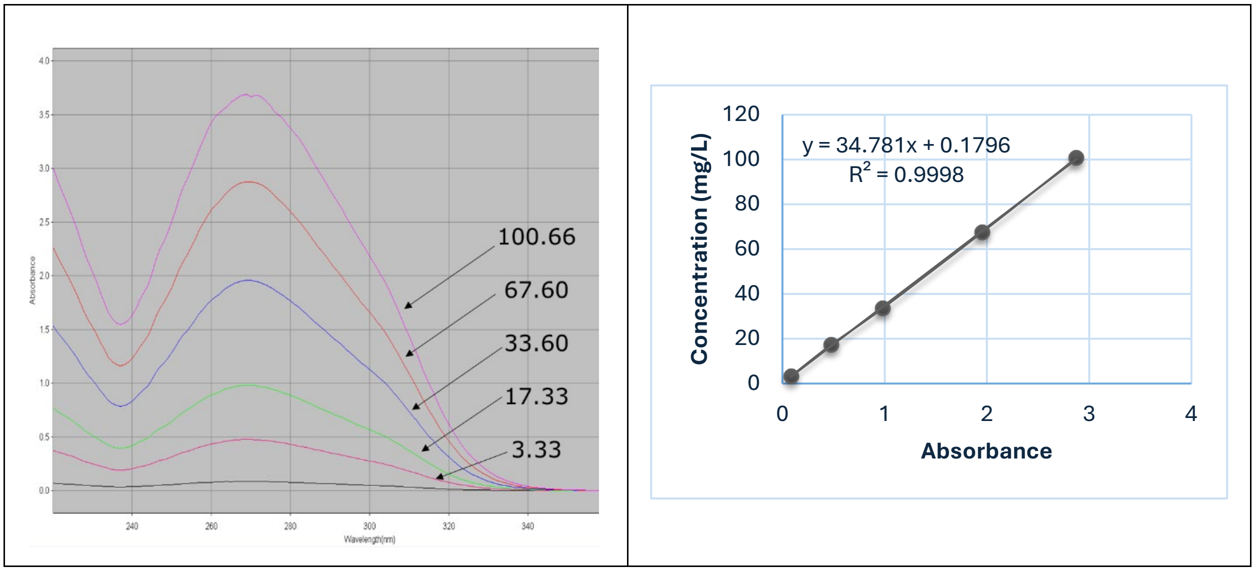

Figure S1. Micafungin standards with concentrations between 3.33 and 100.66 mg L^{-1} and the corresponding plotted calibration curve.

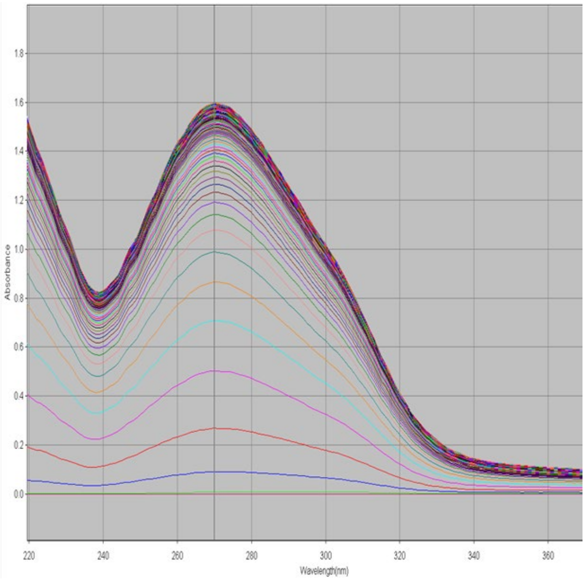

Figure S2. The kinetic time-dependent sustained desorption profile of micafungin recorded in the equipment software.
